# High fidelity hypothermic preservation of primary tissues in organ transplant preservative for single cell transcriptome analysis

**DOI:** 10.1186/s12864-018-4512-5

**Published:** 2018-02-13

**Authors:** Wanxin Wang, Lolita Penland, Ozgun Gokce, Derek Croote, Stephen R. Quake

**Affiliations:** 1 Department of Bioengineering, Stanford University, James H Clark Center, E300, 318 Campus Drive, Stanford, CA 94305 USA; 2 Department of Applied Physics, Stanford University, James H Clark Center, E300, 318 Campus Drive, Stanford, CA 94305 USA; 3 Department of Molecular and Cellular Physiology, Stanford University, Stanford, CA 94305 USA; 4 Chan Zuckerberg Biohub, San Francisco, CA 94158 USA; 5 Institute for Stroke and Dementia Research, Klinikum der Universität München, Ludwig Maximilians Universität LMU, 81377 Munich, Germany

**Keywords:** Single cell RNAseq, Primary tissue preservation without dissociation, Hypothermic preservation, Organ transplant preservation, Organ transplant preservative, Kidney resident immune cells, Transcriptome variability analysis

## Abstract

**Background:**

High-fidelity preservation strategies for primary tissues are in great demand in the single cell RNAseq community. A reliable method would greatly expand the scope of feasible multi-site collaborations and maximize the utilization of technical expertise. When choosing a method, standardizability and fidelity are important factors to consider due to the susceptibility of single-cell RNAseq analysis to technical noise. Existing approaches such as cryopreservation and chemical fixation are less than ideal for failing to satisfy either or both of these standards.

**Results:**

Here we propose a new strategy that leverages preservation schemes developed for organ transplantation. We evaluated the strategy by storing intact mouse kidneys in organ transplant preservative solution at hypothermic temperature for up to 4 days (6 h, 1, 2, 3, and 4 days), and comparing the quality of preserved and fresh samples using FACS and single cell RNAseq. We demonstrate that the strategy effectively maintained cell viability, transcriptome integrity, cell population heterogeneity, and transcriptome landscape stability for samples after up to 3 days of preservation. The strategy also facilitated the definition of the diverse spectrum of kidney resident immune cells, to our knowledge the first time at single cell resolution.

**Conclusions:**

Hypothermic storage of intact primary tissues in organ transplant preservative maintains the quality and stability of the transcriptome of cells for single cell RNAseq analysis. The strategy is readily generalizable to primary specimens from other tissue types for single cell RNAseq analysis.

**Electronic supplementary material:**

The online version of this article (10.1186/s12864-018-4512-5) contains supplementary material, which is available to authorized users.

## Background

Quantitative profiling of transcriptome landscapes at single cell resolution (scRNAseq) has brought new insights in understanding cell types [1, 2], states [3], and interactions [4] in the inherently heterogeneous primary tissues. It, however, has also raised new logistical challenges in the specimen conduit from tissue collection sites to laboratories. The low tolerance to cell damage and RNA degradation in scRNAseq and the less resilient nature of cells in primary tissues make it imperative to process primary specimen immediately after procurement, imposing logistical hurdles especially for collaborations in a multi-institutional setting.

A preservation strategy enabling primary tissue storage and transportation could greatly change the status quo and facilitate collaboration between basic science laboratories and distributed medical centers where tissue is collected. Cryopreservation and chemical fixation have been pursued [5–8], but neither is proven ideal for scRNAseq. In the case of cryopreservation in scRNAseq, a recent study reported tolerable impact of elevated cell death from freeze-thaw on cell lines and specimens with well-represented cell types [6]; another study reported an insufficient recovery and reduced transcriptome complexity for low-abundant and less resilient populations [5]. The susceptibility to handling variations and potential variability in freezing media compositions (e.g. serum) pose challenges for standardization. Furthermore, cryopreservation requires mincing the sample as well as maintenance of temperatures down to − 80 °C. Crosslinking-based chemical fixation, on the other hand, suffers from low recovery of intact mRNA, while alcohol dehydration-based fixation has yet to show high generalizability from cell suspensions to undissociated primary tissues [8]. For efficient isolation of single cells, fixation-based approaches are preferentially done on single-cell suspensions [7, 8], making it a necessity to perform tissue dissociation, usually a critical step for scRNAseq, at tissue collection sites. In fact, aforementioned approaches all require that a multi-step protocol be performed at the collection sites, where experienced personnel are not always available and technical variations can be introduced.

An ideal strategy would avoid drastic physical or chemical changes on the primary specimen and require minimal processing at tissue collection sites. This requirement has much in common with the preservation of organ transplants, which uses hypothermic temperatures to reduce cell metabolism and increase tolerance to insults such as ischemia and hypoxia [9]. Preserving solutions for hypothermic organ preservation hence are designed to address cell-injuring events caused by hypothermia, including ionic imbalance, acidosis, and free radical production [9–11]. Exemplars such as the University of Wisconsin (UW) solution demonstrated high generalizability in preserving post transplant functionality of pancreas (72 h), kidney (72 h), and liver (30 h) [11]. A commercial preparation of such preservative for research use, Hypothermosol-FRS (HTS-FRS), has been increasingly employed in handling primary tissues [12], cells [13–15], and engineered tissue products [16, 17]. Comparative studies done on a spectrum of sample types including human hepatocytes [13], coronary artery smooth muscle cells [14], bone-marrow derived mesenchymal stem cells [15], and mouse hippocampus [12] demonstrated superior efficacy of HTS-FRS in maintaining cell viability compared i) to cell culture media and, in some cases, UW solution in hypothermia as well as ii) to cryopreservation. However, previous studies of the physiologic effects of this approach were mainly limited to viability assays and microscopic examination that interrogate membrane permeability, metabolic activity, cell morphology and surface marker expression. Given that the rapid degradation of RNA could precede the deterioration of these examined parameters [18], previous reports are insufficient to conclude whether preservation fidelity is suitable for scRNAseq.

Here we evaluated hypothermic preservation of primary specimen in HTS-FRS for use in scRNAseq. We used FACS to compare viability of cells recovered from fresh and preserved mouse kidneys, and used scRNAseq analysis to demonstrate the efficacy of the strategy in preserving population heterogeneity, transcriptome integrity, and transcriptome stability of kidney resident immune cells in kidneys undergoing up to 3 days of preservation. This approach enables one to preserve intact primary specimen at 4 °C for periods suitable for long distance transportation of samples and standardization of experimental approaches in expert labs.

## Results

**Fig. 1:**
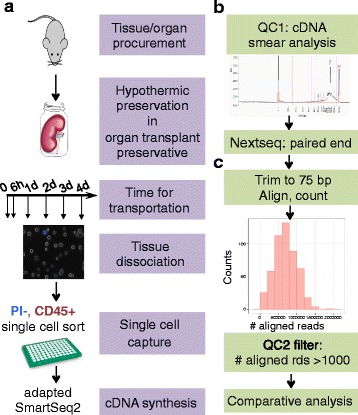
Pipeline design. **a** Schematics and order of preservation, tissue dissociation, single-cell capture, cDNA synthesis, **b** cDNA quality assessment, **c** data processing and quality filtering

We designed our experimental procedure such that preservation preceded dissociation (Fig. 1). Specifically, intact kidneys were preserved for 0, 6 h, or 1–4 days immediately following harvest. After the chosen duration of preservation, we enzymatically digested the tissues into single cell suspension and used FACS to assess overall cell viability. We then used the surface marker Cd45 to enrich for kidney resident immune cells to further evaluate our strategy in the context of scRNAseq, given i) that this population encompasses a diverse spectrum of immune lineages with varying abundance, and ii) the population’s critical role in renal injuries and diseases [19–21].

**Fig. 2:**
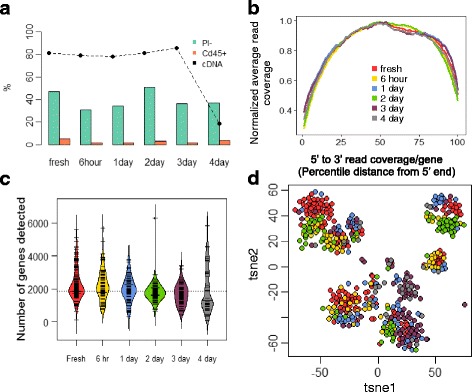
Quality comparison of single cells recovered from fresh and preserved samples (6 h-4d) in terms of (**a**) Overall cell viability (PI-), Cd45+ population abundance, success rates getting sufficient cDNA for sequencing. **b** 5′-3′ read coverage on exons. **c** Distribution of numbers of detected genes over preservation time. **d** tSNE on all detected genes. (Coloring in (**b**), (**c**), (**d**) all follows legend in (**b**))

Over the examined durations of preservation, we observed no notable time-associated reduction for the fractions of propidium iodide negative (PI-) and Cd45+ populations (Fig. 2a, Additional file 1: Figure S1), suggesting that the strategy effectively retained the overall cell viability and the cell surface marker integrity. For each timepoint, PI- and Cd45+ single cells were sorted for scRNAseq analysis. cDNA synthesis on sorted cells gave no notable smearing towards lower fragment sizes for preserved samples (Additional file 1: Figure S2) and comparable success rates in getting sufficient cDNA (≥ 2 ng) between fresh samples and those after up to 3 days of preservation (Fig. 2a). The success rate dropped notably at day 4, despite that the fraction of PI- cells stayed comparable with that of fresh, indicative of the notation that mRNA degradation preceded cell membrane permeabilization in early cell death events [18].

510 single cells with sufficient cDNA level (≥2 ng) were sequenced; 502 (98%) passed quality filtering and were retained for downstream analysis (Fig. 1c). To further evaluate mRNA integrity, we examined 5′ to 3′ read coverage across all exons for each single cell, and observed no more bias towards 3′ in preserved samples than in fresh samples (Fig. 2b), which was further supported by both qualitative inspection of the coverage curves for individual cells (Additional file 1: Figure S3A) and quantitative assessment of the collective skewness of curves for each timepoint (Additional file 1: Figure S3B). In addition, the number of genes detected per cell did not drop noticeably until after 4 days of preservation (Fig. 2c).

**Fig. 3:**
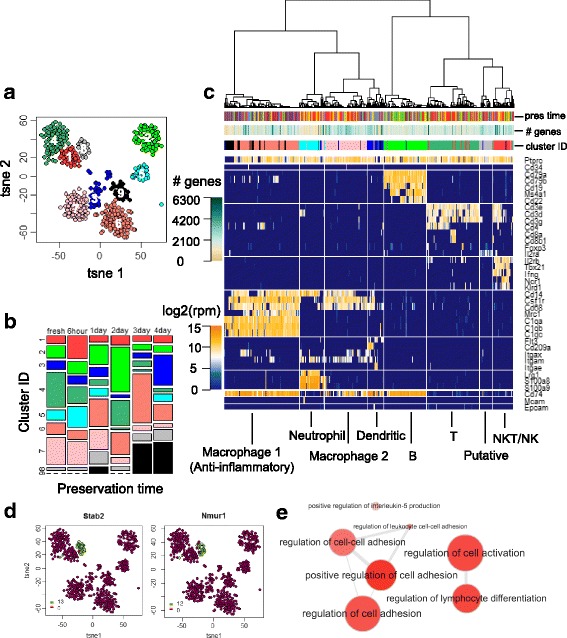
Cell types in kidney resident immune cells and the impact of preservation on cell type heterogeneity. **a** Definition of putative cell clusters on 2d tSNE. **b** Distribution of putative clusters in fresh and preserved tissues (6 h-4d). **c** Identification of putative clusters with known cell types using hierarchical clustering on a canonical panel of genes defining major immune lineages. **d** Expression of representative genes differentially expressed in cluster 8 (color bar shown in log2(rpm + 1)). **e** Hierarchy of enriched ontology terms for differentially expressed genes in putative cluster 8. (All coloring and numbering for cluster ID in (**b**), (**c**) follow those in (**a**); coloring for preservation time follows the legend in Fig. 2b)

We next assessed the impact of preservation on the cell type heterogeneity of kidney resident immune cells. To explore the data in an unbiased manner, we performed dimensional reduction using whole-transcriptome information via t-distributed stochastic neighbor embedding (tSNE). In the resulting 2-dimensional tSNE space (Fig. 2D), single cells formed well-segregated clusters, which we defined into 9 putative clusters computationally (Fig. 3a). Given that it is clear that preservation time is not the driving force for the segregation of the clusters (Fig. 3b), we hypothesized that the source of the segregation was the cell type heterogeneity in kidney resident immune cells, and performed hierarchical clustering on a panel of canonical markers that define major lineages of immune cells. The resulting outcome of the clustering was nearly identical to that observed in the tSNE space (Fig. 3c), confirming our hypothesis.

It is noteworthy that although 7 out of the 9 clusters defined by both methods can be unambiguously identified as known immune populations (Fig. 3c), there are 2 (cluster 8 and 9) that cannot be assigned using classical definitions. Unbiased differential expression analysis on cluster 8 revealed a list of genes that are uniquely expressed in this cluster (Fig. 3d, Additional file 2: Table S1) with enriched ontology terms (Fig. 3E, Additional file 3: Table S2) suggesting it is a putative lymphocyte population that resembles T cells but lacks classical T cell marker expression. Cluster 9, on the other hand, is most likely a low quality/apoptosing macrophage population due to its absence in fresh samples (Fig. 3b), the lack of uniquely expressing markers identified (Additional file 1: Figure S4B), the lack of Cd45/Ptprc expression (Fig. 3c), and the low number of genes expressed (Fig. 3c, Additional file 1: Figure S4A), and hence were excluded in further analysis as a defined population. Each defined population, determined by both methods, is a mixture of cells from fresh tissues and tissues after all examined preservation time (Fig. 3b and c), revealing no preservation associated batch effect. To summarize, the fact that all defined populations were present in fresh tissues and recapitulated over examined duration of preservation validated our definition of cell types in kidney resident immune cells. Moreover, the proposed preservation strategy effectively maintained the heterogeneity of cell types that exist in varying abundance.

**Fig. 4:**
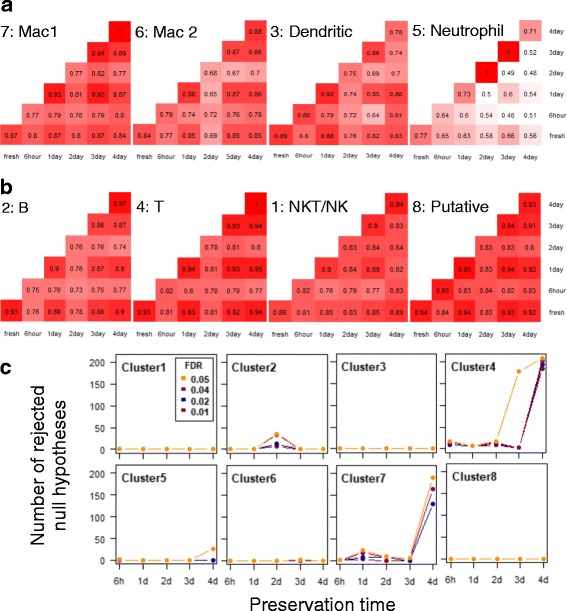
The impact of preservation on the transcriptome profile for each identified cell type. Pair-wise Pearson’s correlation between cells within and across preservation conditions for identified myeloid (**a**) and lymphoid (**b**) populations (Numbers shown are the mediums of each pair of compared distributions; Cell type numbering corresponds to cluster ID in Fig. 3a, and cell type identity follows that in Fig. 3c). **c** Number of rejected null hypotheses in gene differential expression analysis identified between fresh and preserved tissues with incrementing FDR rate

We then evaluated the variability in transcriptomes between cells from fresh and preserved tissues within each of the eight defined cell types. For each cell type, we calculated pair-wise Pearson’s correlation between single cells at two different preservation conditions or within the same condition. As shown in Fig. 4a and b (numbers shown are the medians of distributions of correlations), we did not observe notable decrease in correlations between cells from preserved and fresh tissues within the examined duration of preservation time. Variations in the correlations were dictated, to a greater extent, by the heterogeneity among cells within a given timepoint. The slight deviation in correlations of a given timepoint from fresh is always accompanied by the overall lowered correlations between cells 1) within the timepoint and 2) between the timepoint and any other timepoints in the same cell type, which is likely due to inherent differences between animals.

For all eight clusters, differential expression analysis at a false discovery rate (FDR) ranging from 0.01 to 0.05 revealed no statistically significant differentially expressed genes between fresh and the majority of the timepoints for up to 3 days of preservation (Fig. 4c). Genes identified in the occasional fluctuations observed in cluster 2, 4, 6, and 7 were rather limited in both number and significance level compared to those identified between the given cluster and its nearest neighbor (Additional file 1: Figure S5A). The small groups of genes were dominated by those with 1) borderline adjusted *p*-values or 2) limited fold changes (Additional file 1: Figure S5B, C). The former was particularly prominent for genes identified with FDR = 0.05 at day 3 in cluster 4 (Additional file 1: Figure S5C), which explained the jump in the number of genes from a FDR of 0.04 to 0.05, resulting from an expression burst in singlet cells within a relatively small sample size (Additional file 1: Figure S5D). Moreover, to avoid omitting gene sets that have limited statistical significance but are otherwise biologically relevant, especially as a group, we performed weighted gene set enrichment analysis (GSEA) on all timepoints for each of the 8 clusters. No systematic enrichment of biological processes across timepoints was observed with FDR ≤ 0.05 (Additional file 1: Figure S6, Additional file 4: Table S3, Additional file 5: Table S4, Additional file 6: Table S5).

To further evaluate the variability of genes across timepoints, we performed Breusch-Pagan’s heteroscedasticity test on each mapped gene using preservation time as the independent variable. Genes with null hypothesis rejected at FDR = 0.05 are again limited in number (Additional file 1: Figure S7 and Additional file 7: Table S6). Gene ontology (GO) enrichment analysis revealed no significantly enriched terms for all clusters but cluster 3, which enriched in cell cycle processes (Additional file 8: Table S7). The minimal variability in transcriptome between fresh and preserved cells was further demonstrated by the observation that the top overdispersed genes did not separate preserved from fresh cells as assessed by both dimension reduction and hierarchical clustering (Additional file 1: Figure S8).

**Fig. 5:**
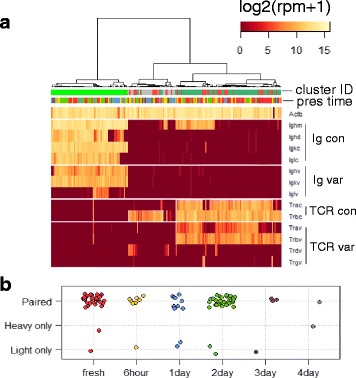
The impact of preservation on B and T cell receptor transcripts. **a** Expression of components of B cell antibodies (Ig) and T cell receptors (TCR) in identified lymphoid populations (con: constant region, var.: variable region). **b** Distribution of success in extracting full-length transcript sequence for heavy and light chains in all identified B cells. (Coloring for preservation time and cluster ID follow that in Fig. 2b and 3a, respectively)

For lymphocyte populations, we specifically examined the transcripts encoding B cell antibodies (Ig) and T cell receptors (TCR) given their essential roles in B and T cell functions and frequent interrogation by single cell studies. As shown in Fig. 5a, expression of key components of Ig and TCR were indistinguishable between fresh and preserved tissues. For B cells, extracting full-length antibody sequence is required for in-depth examination on usage and mutation in variable (V) and joining (J) segments, sequence of complementary-determining region 3 (CDR3), isotypes, as well as affinities. We hence evaluated the success rate in obtaining the information using de novo assembly and the impact of preservation on it. From all B cells identified from fresh and preserved tissues, we were able to identify contigs containing complete variable and constant regions for Ig heavy and light chains (Additional file 9: Table S8). The rate of dropout events where only one chain is identifiable is comparably low between fresh and preserved tissues when statistical power holds (Fig. 5b).

Thus, the strategy introduced minimal variation in the transcriptome landscape of various cell types after up to 3 days of preservation and does not impede proper identifications of cell types and biological pathways of interest.

## Discussion

Hypothermic preservation of primary tissues in organ transplant preservative effectively maintained the viability, transcriptome integrity, and transcriptome profile stability of cells for scRNAseq. Cells recovered from tissues after up to 3 days of preservation demonstrated minimal 3′ bias in the read coverage of exons and comparable cell type heterogeneity compared to cells from freshly harvested tissues.

Resident immune cells from kidneys were used for evaluation in the context of scRNAseq because of their known heterogeneity and the vast interest they have drawn in kidney injuries. We were able to define 8 cell types in this population, supported by their presence in fresh tissue and consistent recapitulation in preserved tissues. Within each population, the preservation strategy did not introduce quantitative perturbations on the overall transcriptome profile, and faithfully preserved Ig and TCR transcripts to a degree that we could assemble full-length transcript sequences for these highly variable genes for higher resolution interrogations.

This approach enables an actionable time window (48–72 h) to be opened up for the transportation of primary specimen from sample collection sites to technology sites through express couriers. The strategy is ideal for scRNAseq also due to its high potential for standardization. Intact tissues can be preserved immediately after excision with minimum intervention. Procedures that are more susceptible to technical variations can then be performed at expert technology sites in a centralized manner, minimizing the introduction of technical noise and variation, especially in highly variable steps such as dissociation. In addition, the preserving solution functions in a serum-free formulation, and hence is free from variations introduced by lot-to-lot difference in serum preparations.

It is noteworthy that tissues and organs from mouse are often small enough that the osmotic gradient is sufficient for passive permeation of the preservative. As the tissue size increases, permeation can be facilitated by vasculature flushing, non-vasculature tissue flushing, and most simply by sectioning the samples into smaller pieces. For whole organs and highly vascularized tissues, flushing via intact vasculatures can be achieved by needle-based flushing or perfusion devices, similar to the practice in preserving organ transplants. For tissues with no significantly visible vasculatures, permeation can be facilitated by directly delivering the preservative to the inner core of the tissue itself through needle-based flushing or sectioning the tissues into smaller pieces. Thanks to the progress made by the organ preservation community, available preservatives such as UW and HTS-FRS already demonstrated high generalizability in preserving functionality in diverse organ types, including pancreas and heart, as well as tissues and organs of various sizes and vasculature complexity, including hair grafts [22], synthetic skin [16], isolated blood vessels [23], tumor biopsy [24], tendon [25], testis [26], and perfused multi-organs during cardiac arrest [27]. We therefore expect that the proposed strategy be readily generalizable to other tissue types for scRNAseq as well as for other procedures such xenograft and organoid generation.

## Conclusions

At single cell resolution, primary tissues after 6 h to 3 days of hypothermic preservation in organ transplant preservative demonstrated similar cell viability, cell type heterogeneity, transcriptome integrity, and transcriptome profile compared to fresh tissues. The strategy is ideal for scRNAseq given its high fidelity and standardizability. The procedure highly resembles the routine handling of specimen in clinics and hence makes it practical to engage clinicians in collaborations, which are essential for the scRNAseq community as well as highly collaborative endeavors such as the Human Cell Atlas.

## Methods

### Mouse kidney isolation, preservation, and dissociation

Single-cell experiments were performed on kidneys of CD1 wild type mice. Mice were housed in filtered cages and all experiments were performed in accordance with approved Institutional Animal Care and Use Committee protocols.

Mice of ~ 3 week old were euthanized by administration of CO_2_. Kidneys were harvested *en bloc* without perfusion and were either dissociated immediately for single cell sort or preserved intact without sectioning in the HTS-FRS solution (BioLifeSolutions) at 4 °C. After 6 h, 1, 2, 3, or 4 days of preservation, intact kidneys were taken out of the HTS-FRS solution for dissociation and further processing. Once taken out, no tissues or tissue sections were put back into preservation for later timepoints. For each timepoint, both (day 0–2) or one-half kidneys (day 3, 4) from the same mouse were pooled for dissociation and sort.

For dissociation, kidneys were minced with a razor blade and dissociated in Liberase DL (Roche) in RPMI 1640 (LifeTechnologies) with horizontal agitation at 180 rpm at 37 °C for 20 min. The resulting single-cell suspension was sequentially passed through a 100 μm, a 70 μm, and a 40 μm strainer (Fisher) and then centrifuged at 300×g for 15 min. Pelleted cells were resuspended in ACK red blood cell lysing buffer (Thermo Fisher), incubated for 5 min, quenched with 1 volume PBS (ThermoFisher) containing 2% FBS (ThermoFisher), centrifuged at 300×g for 5 min, and then resuspended in FACS staining buffer (BD Biosciences).

### Single-cell sorts, cDNA generation, library preparation, and sequencing

Single cells resuspended in the staining buffer were stained with antibody against surface Cd45 (Cd45-FITC, Sony Biotechnology Inc.) on ice for 20 min following manufacture’s protocol, washed twice with the staining buffer, and then incubated in propidium iodide solution (Life Technologies) at room temperature for 10 min. Cell viability was evaluated on FACS (Sony Biotechnology Inc.). Singlet PI^−^ Cd45^+^ cells were index sorted onto pre-chilled 96-well plates containing cell lysis buffer using a Sony SH800 sorter. The plates were vortexed, spun down at 4 °C, immediately placed on dry ice, and then stored at − 80 °C. Single-cell cDNA libraries were generated using procedures adapted from the SmartSeq2 protocol [28]. Briefly, mRNA from single cells in 96-well plates was reverse transcribed using SMARTScribe reverse transcriptase (Clontech), oligo dT, and TSO oligo to generate the first strand cDNA. Resulting cDNA was amplified via PCR (21 cycles) using KAPA HiFi HotStart ReadyMix (KAPA Biosystems) and IS PCR primer. The pre-amplified cDNA was purified using AMPure XP magnetic beads (Beckman Coulter).

Single-cell cDNA size distribution and concentration were analyzed on a capillary electrophoresis-based automated fragment analyzer (Advanced Analytical). Illumina cDNA libraries were prepared for single cells (> 2 ng cDNA generated) using Nextera XT DNA Sample Preparation kit (Illumina) with the single cell protocol provided by Fluidigm. Dual-indexed single-cell libraries were pooled and sequenced in 75 bp or 150 bp pair-ended reads on a Nextseq (Illumina) to a depth of 1–1.5 × 10^6^ reads per cell. CASAVA 1.8.2 was used to separate out the data for each single cell by using unique barcode combinations from the Nextera XT preparation and to generate *.fastq files.

### Processing and analysis of single-cell RNA-seq data

All raw reads in the *.fastq files were trimmed to 75 bp using fastx_trimmer, duplicates-depleted using picard MarkDuplicates, and aligned to Ensembl mouse reference genome GRCm38 using STAR. For every gene in the reference, aligned reads were converted to counts using HTseq and Ensembl GTF under the setting -m intersection-strict \-s no.

Downstream data analysis was performed in R. Prior to analysis, cells with less than 1000 reads were excluded, reducing the dataset from 510 cells to 502 cells. For each cell, counts were normalized to reads per million (rpm) in log2 scale through division by the total number of aligned reads, multiplication by 1 × 10^6^, and conversion to log with base 2. For tSNE, pair-wise distances between cells were calculated using all genes detected. Dimensional reduction was performed using viSNE as implemented in the tsne package [29], and subsequent definition of immune cell lineage clusters were done using hierarchical clustering implemented using Ward’s clustering criterion on the resulting two tSNE dimensions. Differential gene-expression was performed using Wilcoxon’s rank sum test. Statistically significant differentially expressed genes were defined as those with rejected null hypothesis at a given FDR using Benjamini-Hochberg’s procedure [30]; *P*-values were adjusted using Yekutieli and Benjamini’s method with monotonicity enforced [31]. Overdispersion of genes was calculated as CV^2^_i_/CV^2^_e_, where CV^2^_i_ is the squared variation of coefficient of gene i across cells of interest and CV^2^_e_ is the expected squared variation of coefficient given mean [32], fitted using non-ERCC counts. Ontology analysis for uniquely expressed genes associated with putative clusters and heteroscedastic genes was done using enrichment analysis of biological process for *Mus musculus* (www.geneontology.org). Visualization of the hierarchy of the enriched ontology was done using Revigo [33]. GSEA [34] was performed for all timepoints from each of the 8 clusters, using the GSEA software (3.0, GSEAPreranked tool). GO annotations (Mus muculus, Biological process) was used as the gene set database and were converted to .gmt format using GO2MSIG [35]. For each timepoint, all genes with at least 1 mapped read were divided into those that are 1) up-regulated or 2) down-regulated in fresh cells relative to preserved cells based on the sign of log2(fold change). GSEA was then performed on both lists under the default setting, where genes were ranked by –log10(*p*-value) and weighted by the absolute value of log2(fold change).

No thresholds were used to exclude genes from analysis. Breusch-Pagan test was performed using the R package bptest, where preservation time was used as the independent variable.

### Assembly of B cell antibody heavy and light chains

Full length, paired immunoglobulin heavy and light chain sequences from single B-cells were assembled and annotated by first trimming raw reads with fqtrim, followed by full transcriptome assembly with Bridger [36]. Immunoglobulin contigs identified through the presence of a heavy or light chain constant region sequence were then annotated using IgBLAST [37]. Variable (V), diversity (D), joining (J), and complementarity determining region (CDR) calls were extracted from the IgBLAST output using Change-O [38].

## Additional files

Additional file 1: **Figure S1.** Overall viability and abundance of Cd45+ cells across different durations of preservation time. At the bottom of each panel, the percentage in white was calculated as: counts of PI- events / counts of all events for the top panels, and counts of Cd45+ events / counts of all events for the bottom panels. Raw counts were shown in the fractions. **Figure S2.** cDNA concentration and smearing assessed via fragment analysis for single Cd45+ cells from mouse kidneys after different durations of preservation presented as (A) electrophoresis traces and (B) gel image. **Figure S3.** Genebody coverage of Cd45+ single cells from mouse kidneys after different durations of time. (A) 5′-3′ read coverage on exons. (B) Distribution of skewness of 5′-3′ read coverage on exons. **Figure S4.** Identification of cell types in Cd45+ single cells from mouse kidneys. (A) Number of genes detected cast on 2d tSNE. (B) Uniquely expressing genes identified for each putative cell clusters. (Coloring of cluster ID follows that in Fig. 3A.) **Figure S5.** genes rejected by null hypothesis (DE genes) at FDR = 0.05 between fresh and preserved tissue in cluster 2, 4, 6, 7. (A) Number of DE genes identified between each of the eight identified cell types and its nearest neighbor (defined in Fig. 3A) with incrementing FDR. (B) Volcano plots for DE gene at FDR = 0.05 between fresh and preserved tissue identified in the given cluster (blue) and DE genes identified in (A) for the same cluster (black). (C)(D) DE genes at FDR = 0.05 in cluster 4 between fresh and day 3 tissues. (Cluster ID and color for time followed that in Fig. 3A.) **Figure S6.** Number of gene sets enriched with FDR q value≤0.05 for genes that are (A) upregulated or (B) downregulated in cells from fresh tissues compared to those from preserved tissues. **Figure S7.** Number of genes with rejected null hypothesis by the Breusch-Pagan test at incrementing FDR for each identified cell cluster. **Figure S8.** Evaluation of gene expression variation between cells from fresh and preserved tissues via (A) dimension reduction on incrementing number of overdispersed genes (B) hierarchical clustering on top 500 over-dispersed genes (cluster ID follows that in Fig. 3A, cell clustering follows Fig. 2D). (PDF 6690 kb)

Additional file 2: **Table S1.** Differentially expressed genes in the putative cluster (cluster 8). (CSV 1 kb)

Additional file 3: **Table S2.** GO ontology of genes differentially expressed in the putative cluster (cluster 8). (CSV 1 kb)

Additional file 4: **Table S3.** Gene sets enriched with FDR q value ≤0.05 from GSEA. (CSV 732 bytes)

Additional file 5: **Table S4.** Output (top 20 hit) from GSEA on genes with positive log2(fold change) between cells from fresh and preserved tissues. (XLSX 108 kb)

Additional file 6: **Table S5.** Output (top 20 hit) from GSEA on genes with negative log2(fold change) between cells from fresh and preserved tissues. (XLSX 104 kb)

Additional file 7: **Table S6.** Genes with null hypothesis rejected at FDR = 0.05 by Breusch-Pagan test. (XLSX 15 kb)

Additional file 8: **Table S7.** Gene ontology enrichment of genes from cluster3 with null hypothesis rejected at FDR = 0.05 by Breusch-Pagan test. (CSV 668 bytes)

Additional file 9: **Table S8.** Detailed annotation on assembled full-length transcripts for antibody heavy and light chains in all identified B cells. (CSV 8 kb)

## Abbreviations

CDR: Complementarity determining region
D: Diversity
FDR: False discovery rate
GO: Gene ontology
GSEA: Gene set enrichment analysis
HTS-FRS: Hypothermosol-FRS
Ig: Immunoglobulin/antibody
J: Joining
PI-: Propidium iodide negative
scRNAseq: Single cell RNAseq
TCR: T cell receptor
tSNE: t-distributed stochastic neighbor embedding
UW: University of Wisconsin solution
V: Variable
