# Supplementary material for: High fidelity hypothermic preservation of primary tissues in organ transplant preservative for single cell transcriptome analysis
Source: BMC Genomics. 2018 Feb 13;19:140. doi: 10.1186/s12864-018-4512-5 (PMC5811979; doi:10.1186/s12864-018-4512-5)
Supplement: Supplementary file 1 — Figure S1. Overall viability and abundance of Cd45+ cells across different durations of preservation time. At the bottom of each panel, the percentage in white was calculated as: counts of PI- events / counts of all events for the top panels, and counts of Cd45+ events / counts of all events for the bottom panels. Raw counts were shown in the fractions. Figure S2. cDNA concentration and smearing assessed via fragment analysis for single Cd45+ cells from mouse kidneys after different durations of preservation presented as (A) electrophoresis traces and (B) gel image. Figure S3. Genebody coverage of Cd45+ single cells from mouse kidneys after different durations of time. (A) 5′-3′ read coverage on exons. (B) Distribution of skewness of 5′-3′ read coverage on exons. Figure S4. Identification of cell types in Cd45+ single cells from mouse kidneys. (A) Number of genes detected cast on 2d tSNE. (B) Uniquely expressing genes identified for each putative cell clusters. (Coloring of cluster ID follows that in Fig. 3A.) Figure S5. genes rejected by null hypothesis (DE genes) at FDR = 0.05 between fresh and preserved tissue in cluster 2, 4, 6, 7. (A) Number of DE genes identified between each of the eight identified cell types and its nearest neighbor (defined in Fig. 3A) with incrementing FDR. (B) Volcano plots for DE gene at FDR = 0.05 between fresh and preserved tissue identified in the given cluster (blue) and DE genes identified in (A) for the same cluster (black). (C)(D) DE genes at FDR = 0.05 in cluster 4 between fresh and day 3 tissues. (Cluster ID and color for time followed that in Fig. 3A.) Figure S6. Number of gene sets enriched with FDR q value≤0.05 for genes that are (A) upregulated or (B) downregulated in cells from fresh tissues compared to those from preserved tissues. Figure S7. Number of genes with rejected null hypothesis by the Breusch-Pagan test at incrementing FDR for each identified cell cluster. Figure S8. Evaluation of gene expression variation betwee [file 12864_2018_4512_MOESM1_ESM.pdf]

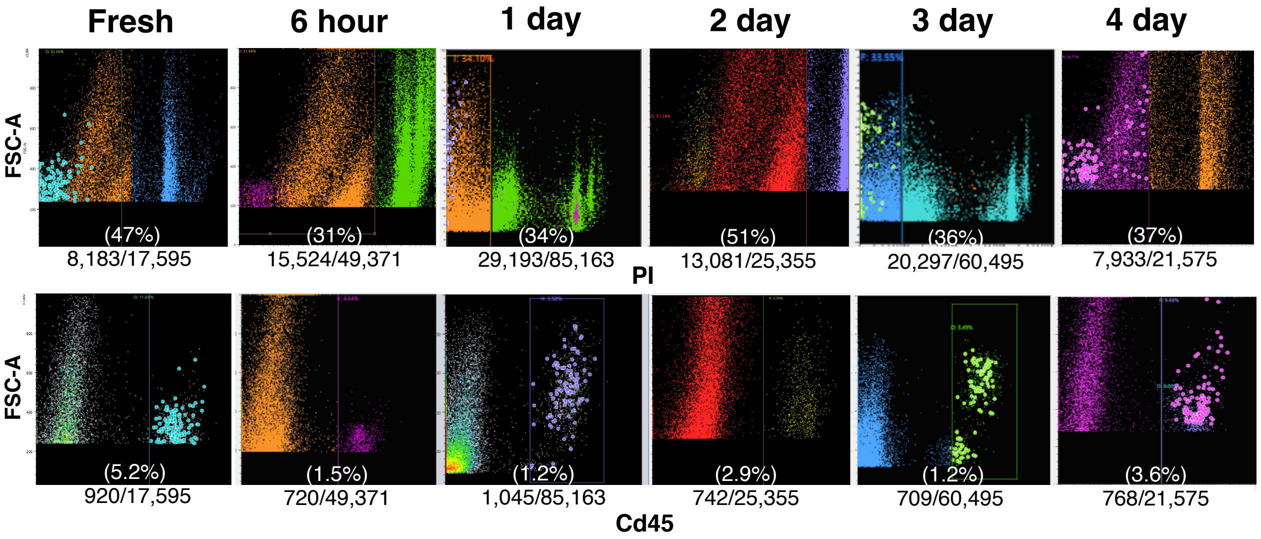

Figure S1.

Overall viability and abundance of Cd45+ cells across different durations of preservation time. At the bottom of each panel, the percentage in white was calculated as: counts of PI- events / counts of all events for the top panels, and counts of Cd45+ events / counts of all events for the bottom panels. Raw counts were shown in the fractions.

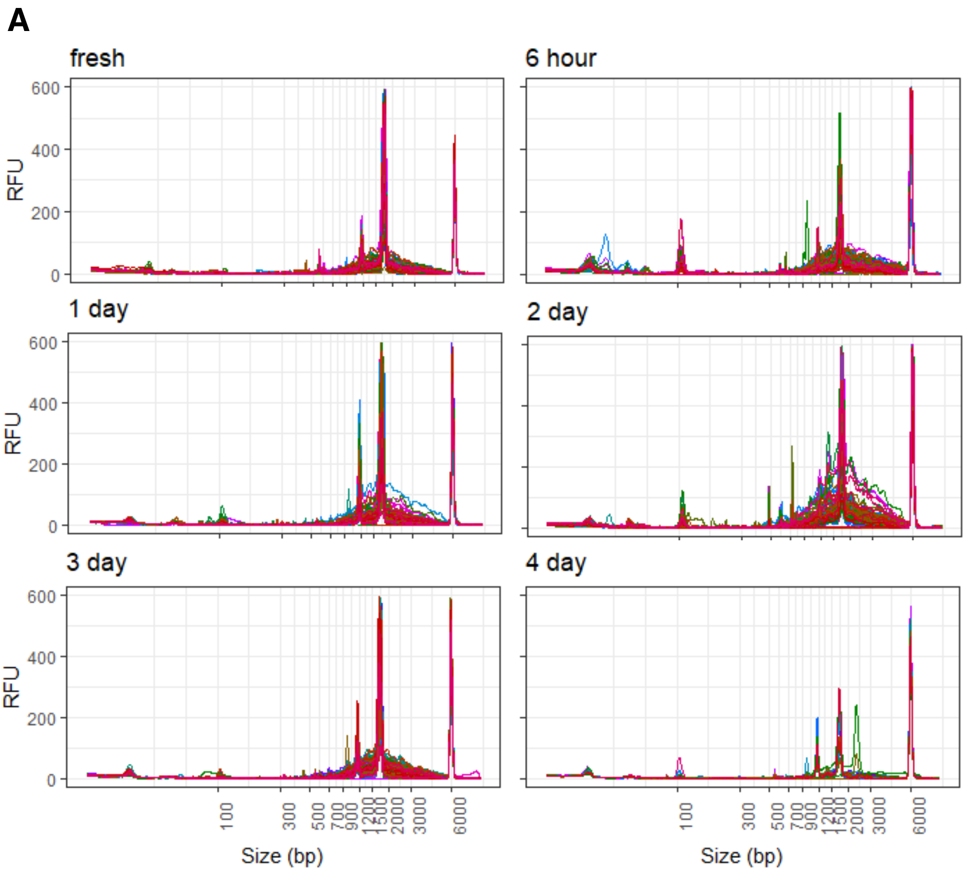

Figure S2.
cDNA concentration and smearing assessed via fragment analysis for single Cd45+ cells from mouse kidneys after different durations of preservation presented as (A) electrophoresis traces and (B) gel image.

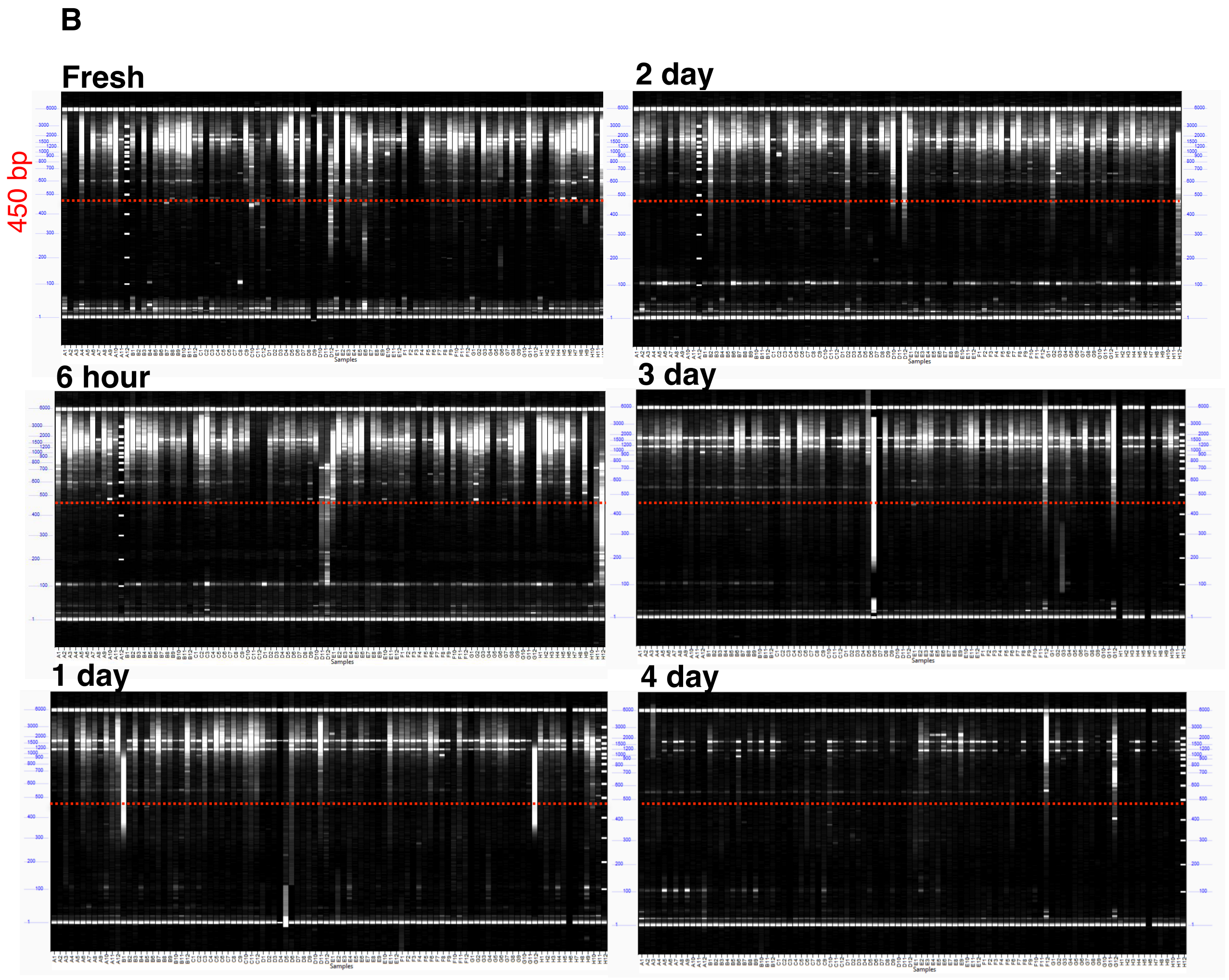

Figure S2. (Continued)

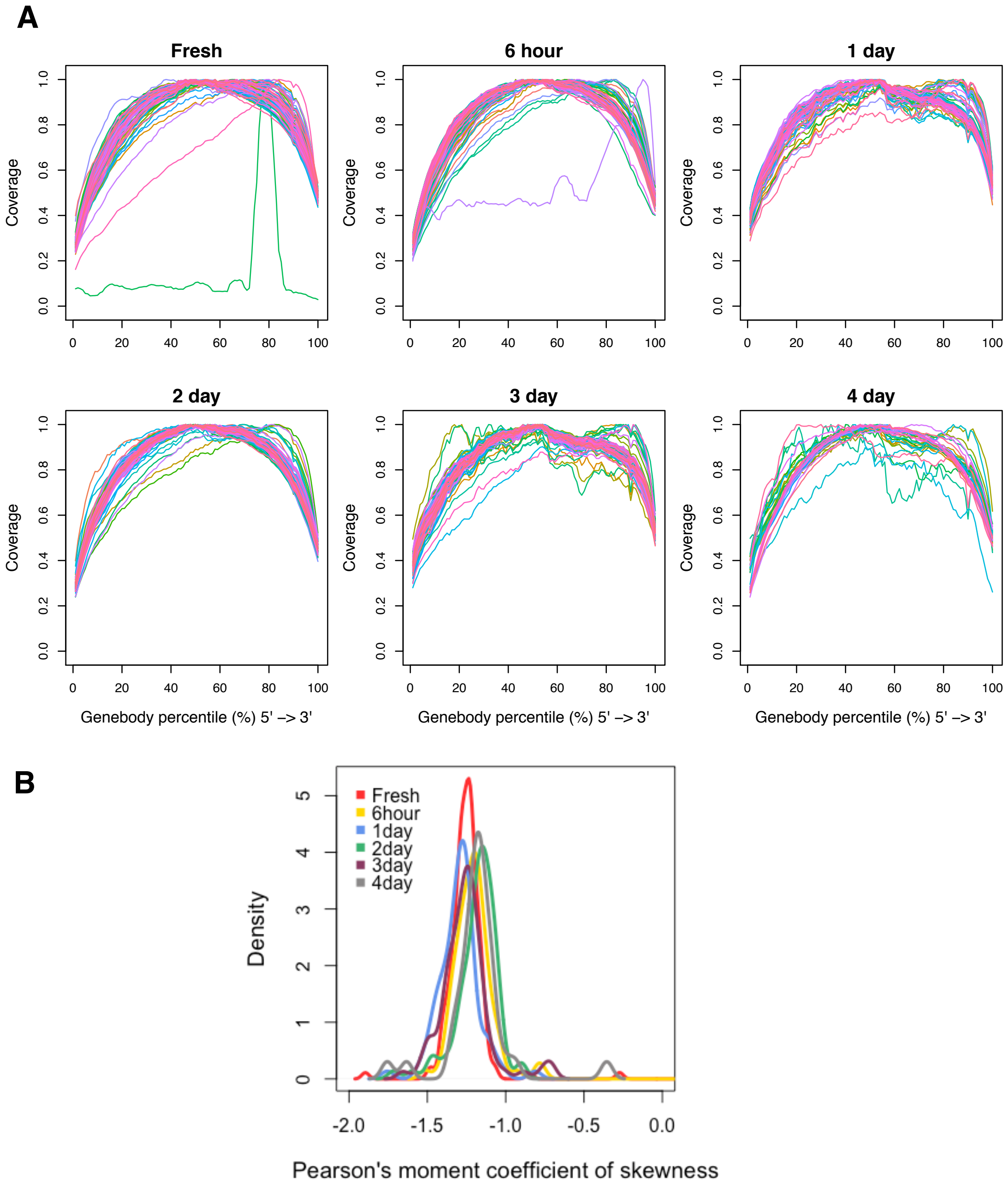

Figure S3.

Genebody coverage for Cd45+ single cells from mouse kidneys after different durations of time. (A) 5’-3’ read coverage on exons. (B) Distribution of skewness of 5’-3’ read coverage on exons.

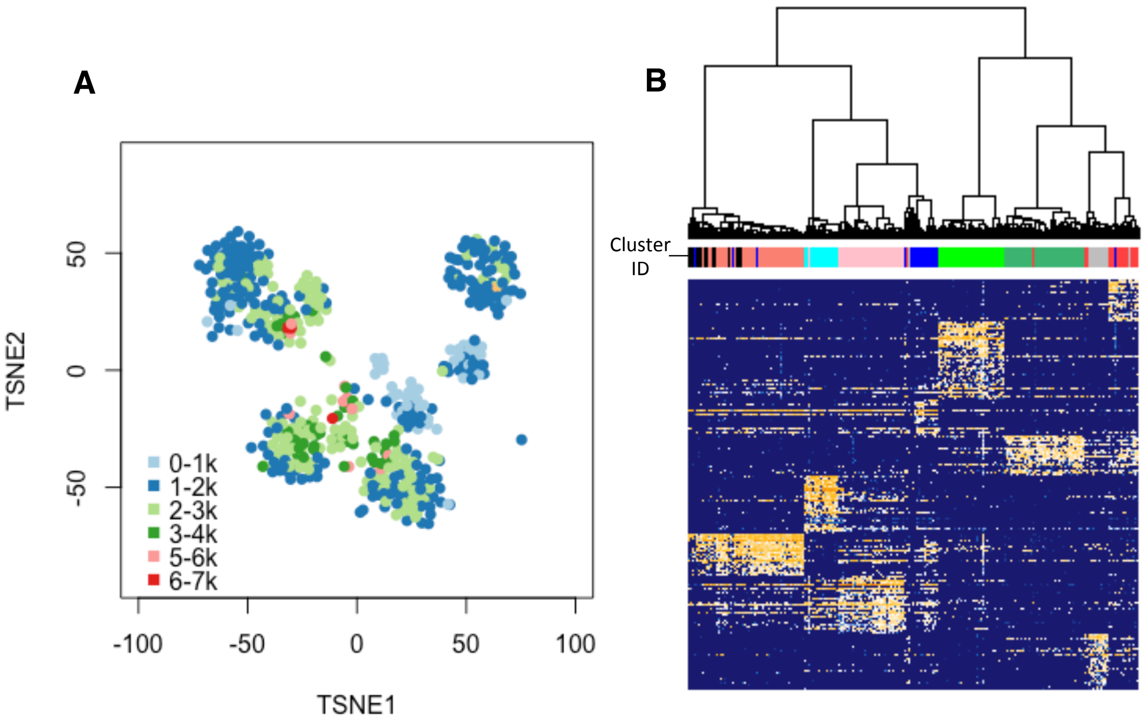

Figure S4.
Identification of cell types in Cd45+ single cells from mouse kidneys. (A) Number of genes detected cast on 2d tSNE. (B) Uniquely expressing genes identified for each putative cell clusters. (Coloring of cluster ID follows that in Fig 3A.)

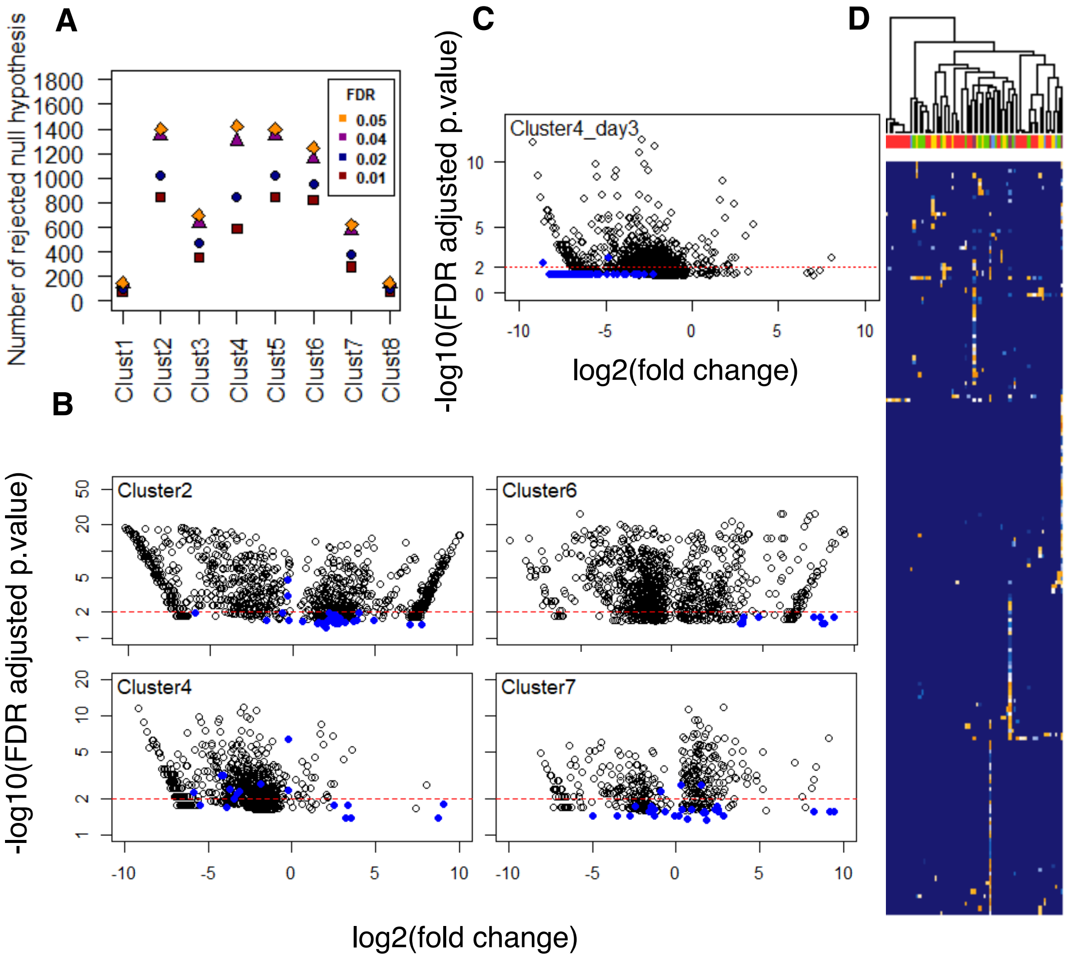

Figure S5.
Genes rejected by null hypothesis (DE genes) at FDR=0.5 between fresh and preserved tissue in cluster 2, 4, 6, 7. (A) Number of DE genes identified between each of the eight identified cell types and its nearest neighbor (defined in Fig 3A) with incrementing FDR. (B) Volcano plots for DE gene at FDR=0.05 between fresh and preserved tissue identified in the given cluster (blue) and DE genes identified in (A) for the same cluster (black). (C)(D) DE genes at FDR=0.05 in cluster 4 between fresh and day 3 tissues. (Cluster ID and color for time followed that in Fig 3A.)

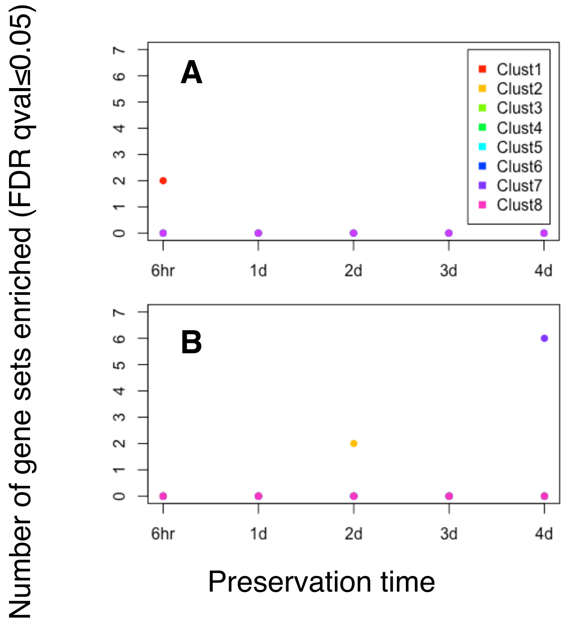

Figure S6.
Number of gene sets enriched with FDR≤0.05 for genes that are (A) upregulated or (B) downregulated in cells from fresh tissues compared to those from preserved tissues.

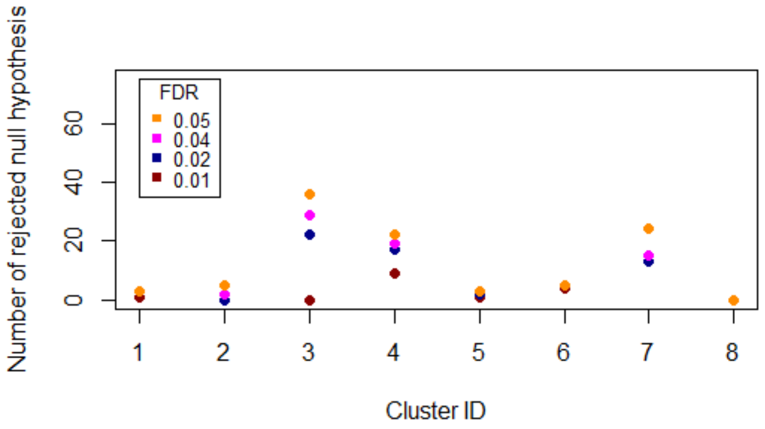

Figure S7.
Number of genes with rejected null hypothesis by the Breusch-Pagan test at incrementing FDR for each identified cell cluster.

A

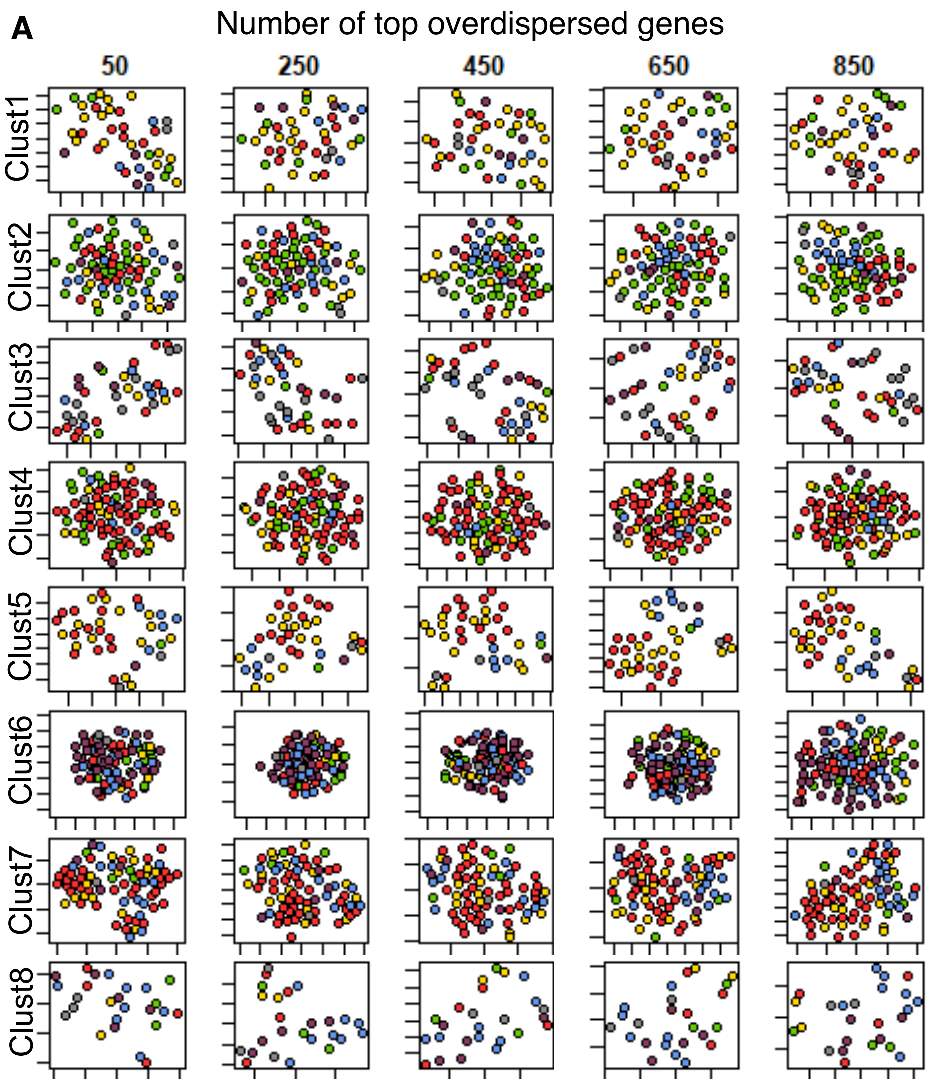

Figure S8.
Evaluation of gene expression variation between cells from fresh and preserved tissues via (A) dimension reduction on incrementing number of overdispersed genes (B) hierarchical clustering on top 500 over-dispersed genes (cluster ID follows that in Fig 3A, cell clustering follows Fig 2D).

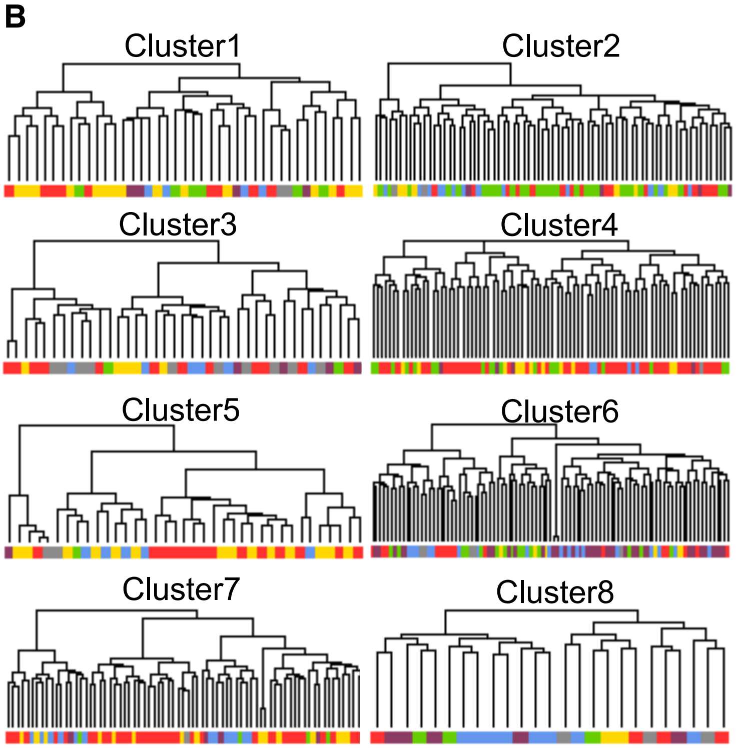

Figure S8. (continued)
